# Pneumatic retinopexy for primary rhegmatogenous retinal detachment: from a clinical trial to the real-life experience

**DOI:** 10.1186/s12886-024-03559-7

**Published:** 2024-07-16

**Authors:** Danilo Iannetta, Nicola Valsecchi, Alessandro Finzi, Rodolfo Mastropasqua, Rajeev H. Muni, Luigi Fontana

**Affiliations:** 1 Ophthalmology Unit, Dipartimento di Scienze Mediche e Chirurgiche, Alma Mater Studiorum University of Bologna, Bologna, Italy; 2 IRCCS Azienda Ospedaliero-Universitaria di Bologna, Via Pelagio Palagi 9 Bologna,, Bologna, Postal code, 40138 Italy; 3 University of Rome La Sapienza Department of Organs of Sense, Rome, Italy; 4 Ophthalmology Clinic, Department of Medicine and Science of Ageing, University G. D’Annunzio Chieti-Pescara, Chieti-Pescara, Italy; 5 Department of Ophthalmology, St. Michael’s Hospital/Unity Health Toronto, Toronto, ON Canada; 6 Department of Ophthalmology and Vision Sciences, University of Toronto, Toronto, ON Canada

**Keywords:** Pneumatic Retinopexy, Rhegmatogenous Retinal detachment, Real-world outcomes, Surgical success, Complications

## Abstract

**Background:**

To report real-world outcomes of patients with primary Reghmatogenous Retinal Detachment (RRD) treated with Pneumatic Retinopexy (PnR) according to the indications of the Pneumatic Retinopexy versus Vitrectomy for management of Primary Rhegmatogenous Retinal Detachment Outcomes Randomized Trial (PIVOT) trial.

**Methods:**

Multicenter, retrospective study. Patients treated with PnR for RRD between 2021 and 2023 and a follow-up of at least 6 months were included. Single-procedure anatomical success, final anatomical success, complications, causes of failures, best corrected visual acuity (BCVA) after surgery, and the vision-related quality of life using the 25-Item National Eye Institute Visual Function Questionnaire (NEI VFQ-25) were reported.

**Results:**

A total of 76 eyes of 76 patients were included. Mean age was 60 ± 8.1 years. Primary anatomic reattachment was achieved by 84.3% of patients and final anatomical reattachment after pars plana vitrectomy was obtained in 100% of patients. BCVA improved from 0.32 (20/40) to 0.04 (20/20) logMar (*p* < 0.001) at 6 months. The main cause of failure was related to the presence of additional (likely missed) retinal breaks (66.6% of cases). Also, primary PnR failure was more frequent in eyes of patients with older age, macular involvement, worse baseline BCVA, greater extent of the RRD, and increased duration from diagnosis to treatment. Overall, the mean NEI-VFQ 25 composite score was 93.9% ± 6.4 at 6 months.

**Conclusions:**

The criteria of the PIVOT trial can be applied to real-world scenarios in the decision-making process for the treatment of primary RRD, with excellent anatomical and functional outcomes.

## Introduction

Rhegmatogenous retinal detachment (RRD) is the most common form of retinal detachment, and it develops when a retinal break in the neurosensory retina (NSR) allows the entry of fluid from the vitreous cavity into the subretinal space, resulting in a separation of the NSR from the retinal pigment epithelium (RPE) [1]. The incidence of RRD ranges between 6.3 and 17.9 cases per 100,000 population [2]. Currently, there are three different methods of repairing RRD: pneumatic retinopexy (PnR), scleral buckling (SB), and pars plana vitrectomy (PPV). In 1985, Dominguez described PnR as an outpatient procedure for RRD without conjunctival incision [3, 4]. PnR was subsequently popularized by Hilton & Grizzard [5]. Later, a prospective, multicenter, randomized controlled trial compared PnR with SB for RRD, demonstrating superior visual acuity (VA) with PnR at 6 months and 2 years after surgery, including those patients whose primary PnR failed, with no significant difference in primary reattachment rates [6, 7]. Moreover, the study reported a lower rate of morbidity and a faster rehabilitation for patients [6].

In 2018, the PIVOT trial compared two groups of patients with primary RRD who were randomized to either PnR or PPV. Macula-on and macula-off patients were assigned to the intervention groups through stratified randomization and were treated within 24 and 72 h, respectively [8]. They included patients with RRD who exhibited a single retinal break or a cluster of breaks in the detached retina within 1 clock hour above the 8- and 4-o’clock meridians, as well as any number, location, and size of retinal breaks or lattice degeneration in the attached retina. The main results of the PIVOT trial were that primary anatomic reattachment at 12 months was achieved by 80.8% of patients who underwent PnR versus 93.2% undergoing PPV and that 98% of patients achieved secondary anatomic reattachment in both groups. Also, the PIVOT trial demonstrated that patients treated with PnR had superior visual acuity, a lower rate and severity of vertical metamorphopsia and higher NEI-VFQ-25 scores (in the first 6 months) compared to patients treated with PPV, suggesting that PnR should be considered as a first line treatment in those patients that fulfill the inclusion criteria for the PIVOT trial [8, 9].

Time from presentation and diagnosis to surgery is an important predictor of future visual acuity outcomes, particularly in patients with macula-off retinal detachments [10–13]. A recent meta-analysis reported a better final visual acuity in patients with fovea-off RRD treated within 72 h, and in patients with fovea-on RRD treated within 24 h compared to patients treated later [12]. Experimental studies showed that hypoxia induced by retinal detachment causes a gradual death of the photoreceptor cells, contributing to the poor vision outcome [14]. Also, several studies reported that the outer retinal layer damages were more commonly observed in patients with macula-off RRD after surgery who had a longer duration of macular detachment [15, 16].

The promptness of surgical repair may partially explain the excellent outcomes reported in the PIVOT study, although on average patients in the PPV group in the PIVOT trial had surgical management within 24 h (within 24 h for fovea-on RRD and within 72 h for fovea-off RRD as per trial protocol) far exceeding real-life operating room access. However, the patients were managed within the context of a randomized trial and it is possible that the primary reattachment rates reported may differ from that achievable in a real-world setting. Furthermore, PnR is infrequently used for the treatment of RRD in Italy. Therefore, the aim of the present study was to report the real-world outcomes in Italy of patients treated with PnR selected according to the inclusion criteria of the PIVOT trial.

## Materials and methods

### Study population and clinical assessment

Retrospective data of patients treated for primary RRD with PnR between January 2021 and March 2023 at the Unit of Ophthalmology, IRCSS University of Bologna and at the Ophthalmic Clinic of the G. d’Annunzio University of Chieti-Pescara, Italy were collected. The study was conducted in accordance with the principles of the Declaration of Helsinki. Written informed consent was obtained from all subjects included in the study.

The primary outcome of the study was to assess the primary anatomic reattachment rate and the final anatomic reattachment rate at 6 months in patients treated with PnR. The secondary outcome of the study was to assess the complications, causes of failures, the best corrected visual acuity (BCVA), and the vision-related quality of life assessed with the National Eye Institute Visual Function Questionnaire − 25 (NEI-VFQ 25) in patients treated with PnR at 6 months.

In the present study, we included eyes of patients treated with PnR according to the inclusion and exclusion criteria of the PIVOT trial (Table 1 at the end of the document text file page 16), a follow-up of at least 6 months, complete information regarding the clinical data and concomitant comorbidities, and patient’s compliance with post-operative visits. No maximum limit age was established. Also, we excluded patients with a diagnosis of other retinal diseases (vascular retinal diseases, chorioretinal disease, inherited retinal dystrophies), glaucoma and other optic nerve diseases, history of uveitis, corneal opacities, and systemic diseases potentially affecting the results of the study.

**Table 1:** Inclusion and exclusion criteria for PnR according to the PIVOT study

**Inclusion criteria**
- Single break or a group of small breaks no larger than 1 o’clock hour in detached retina
- All breaks in the detached retina to lie above the 8 and 4 o’clock meridian
- Breaks or lattice degeneration in attached retina at any location
**Exclusion criteria**
- Inferior breaks in detached retina - PVR grade B or worse - Significant media opacities - < 18 years old - Mental incapacity or physical inability to posture post-operatively

Legend: PVR = proliferative vitreoretinopathy

Patients were treated within 48 h from diagnosis in both fovea-on and fovea-off cases. Formal study visits and observations took place at baseline, 1 day, 1 week, and 1, 3, and 6 months after surgery. Any number of additional visits were performed as required for that patient’s care. Clinical examination and measurement of BCVA took place at every visit. Subjective visual function was assessed at 6 month visits using the NEI VFQ-25 ^17^ The NEI VFQ-25 is a vision-specific quality-of-life instrument composed of 12 subscales: general health, general vision, ocular pain, near activities, distance activities, social functioning, mental health, role difficulties, dependency, driving, color vision, and peripheral vision. Each scale consists of a minimum of 1 and maximum of 4 items. Most items are scored using a 5-point or 6-point response scale. A standard algorithm was used to calculate the scale scores, which have a possible range of zero to 100. Eleven of 12 scale scores (excluding the general health item) were averaged to yield a composite score, as previously described [17].

### Surgical intervention

At the baseline evaluation in the office, all patients underwent a thorough scleral-depressed peripheral retinal examination in order to identify the retinal breaks. When inclusion and exclusion criteria were met, PnR was performed according to the surgical indications of the PIVOT trial. In cases of lattice degenerations or retinal breaks in the attached retina, laser retinopexy was applied before surgery in the office. PnR procedures were carried out under local (sub-conjunctival) anesthesia in the operating room, and all surgeries were conducted by three surgeons (DI, RM, AF). Breaks in the detached retina were treated with cryotherapy before gas injection or laser retinopexy 24 to 48 h after gas injection, with additional laser retinopexy applied at any point according to surgeon discretion. An anterior chamber paracentesis was used to express as much fluid as safely possible, followed by injection of 0.6 ml of 100% sulphur hexafluoride (SF6). Most patients underwent the so-called steamroller maneuver to expedite retinal reattachment, by using the buoyant force of the gas bubble to displace subretinal fluid (SRF) through the retinal break and to protect the macula from displaced SRF. Subsequent supplementary gas injection or laser application were performed at the surgeon’s discretion.

### Statistical analysis

Normality was tested with the Shapiro–Wilk test, and parametric tests were used in the statistical analysis.

Values were reported with mean and standard deviations. The T-Test was used to compare pre-operative clinical assessment parameters between patients with and without primary anatomic reattachment, and to compare vision related quality of life scores between patients with fovea-on and fovea-off RRD. Also, the T-Test was used to compare extent of retinal detachment between patients with fovea-on and fovea-off RRD. Paired T-test was used to compare BCVA before and at the 6-month follow-up. Fisher’s exact test was used to measure the differences between two categorical variables. *P* values < 0.05 were considered statistically significant. Statistical analysis was performed using IBM Statistical Package for Social Sciences version 26.

## Results

### Demographic characteristics

A total of 76 eyes of 76 patients were included in the study. Demographic data of the patients are reported in Table 2 (at the end of the document text file page 17). Patients were treated in a mean of 22.7 ± 12 h from the diagnosis. In the fovea-on group, PnR was performed within 22 ± 11 h from the diagnosis, whereas in the fovea-off group PnR was performed within 24 ± 14 h from the diagnosis. In the present study, we did not detect any lattice degenerations or retinal breaks in attached retina before PnR. Therefore, pre-operative laser retinopexy was never performed.

**Table 2:** Demographic data of patients treated with PnR are reported

Pre and post-operative characteristics (*n* = 76)	
Age, mean ± SD	60 ± 8.1
Female, number (%)	40 (52.6%)
FOVEA ON, number (%)	52 (68.4%)
Phakic patients, number (%)	68 (89.5%)
Baseline BCVA LogMar, mean (min - max)	0.3 (1.3–0)
Time to treatment (hours), mean (min - max)	22.7 (3–48)
Post-op laser photocoagulation, number (%)	64 (84.2%)
Number of retinal breaks, mean ± SD	1.4 ± 0.5
Extension of retinal detachment (hours), mean ± SD	3.4 ± 1.6
Single procedure anatomical success rate, number (%)	64 (84.2%)
Final anatomical reattachment, number (%)	76 (100%)

### Primary outcome

The primary anatomic reattachment rate was 84.3% (64/76) at 6 months. None of these patients required a second gas bubble injection. Those patients who were not successfully treated with PnR underwent a secondary PPV in combination with phacoemulsification and intraocular lens (IOL) implantation. Overall, final anatomical reattachment was achieved in all patients treated (100%, 76/76).

### Secondary outcomes

**Fig. 1:**
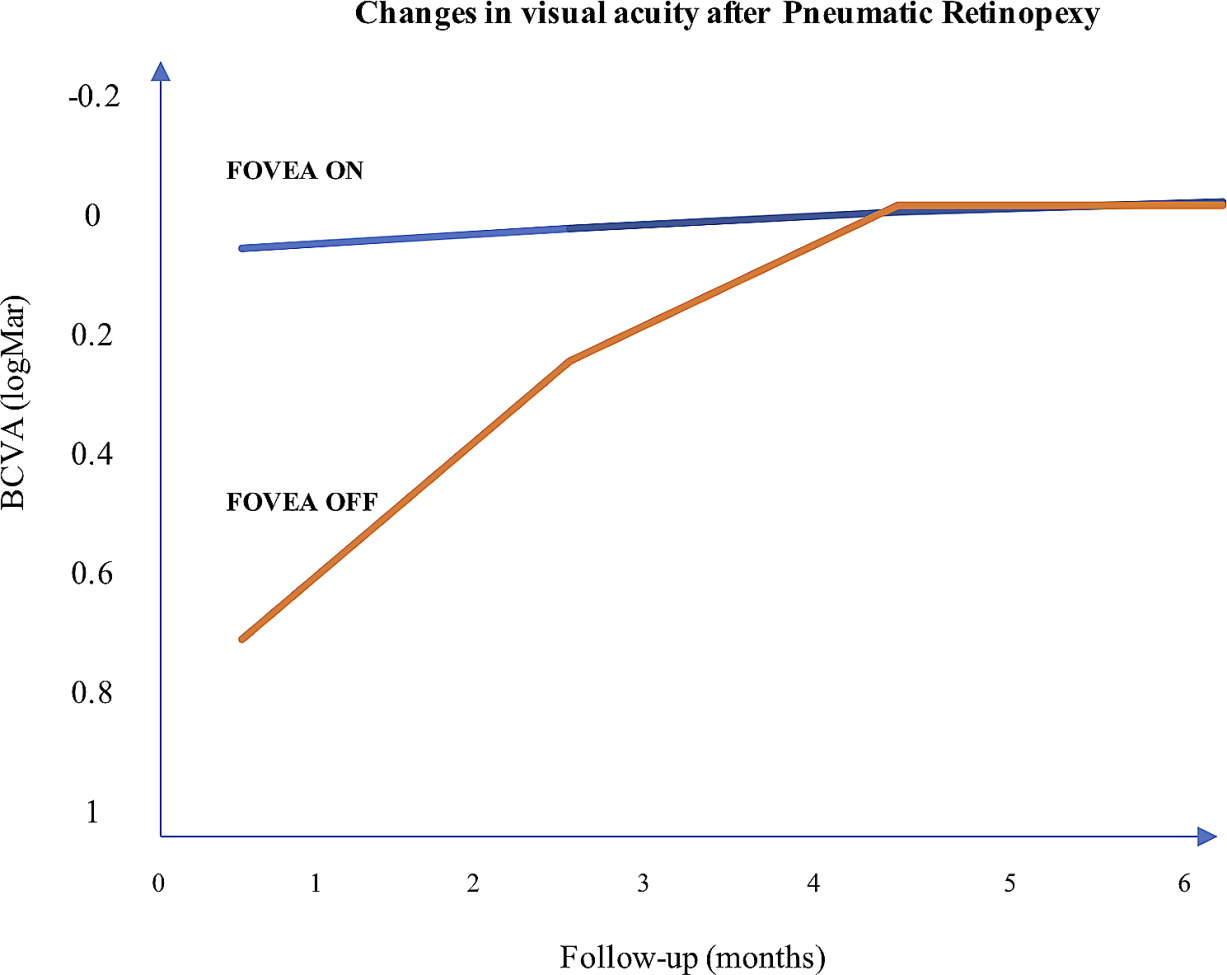
Changes in visual acuity in patients with macula involvement at six months

Overall, BCVA improved from 0.32 (20/40) to 0.04 (20/20) LogMar (*p* < 0.001) at 6 months. BCVA in the fovea-off group improved from 0.85 (20/120) to 0.04 (20/20) LogMar at six months (*p* = 0.005), and it remained stable in the fovea-on group from 0.10 (20/25) to 0.05 (20/20) at 6 months (*p* = 0.764) (Fig. 1). The main cause of failure was related to the presence of additional (likely missed) retinal breaks (66.6% of cases, 8/12), whereas failure of retinal reattachment for poor compliance in maintaining position was observed in 33.3% (4/12) of cases. In the present study, there were no minor and major complications among all patients treated (Table 3 at the end of the document text file page 18). No additional visits were performed in patients with primary anatomic reattachment after PnR. Those patients who presented surgical failure after PnR and who required secondary PPV received a mean of 3.2 ± 2.1 additional visits. Surgical failure was significantly more frequent in patients with older age, foveal involvement, increased extent of the retinal detachment, worse baseline BCVA and increased time to treatment (Table 4 at the end of the document text file page 19). Patients who presented surgical failure secondary to additional retinal breaks presented an increased extension of the retinal detachment and a reduced visual acuity at the baseline assessment compared to patients with failure secondary to poor compliance in maintaining position (6.5 ± 1.6 vs. 4.0 ± 0.1, *p* = 0.012; 1.3 ± 0.1 vs. 0.7 ± 0.1, *p* < 0.001). On the other hand, poor compliance in maintaining position did not differ according to the age of patients or the time from diagnosis to treatment (73 ± 0.1 vs. 71.5 ± 3.7 years, *p* = 0.452; 36.2 ± 3.2 vs. 26.5 ± 2.8 h, *p* = 0.157). Regarding the assessment of the subjective visual function using the NEI VFQ-25 at 6 months, the mean composite score was 93.96% (median = 96.1 (IQR = 92.8–97.7)) at 6 months (Table 5 at the end of the document text file page 16).

**Table 3:** Causes of failure and complications

Causes of failure (*n* = 12)	*n* (%)
New and missed retinal breaks	8 (66.6%)
Failure of the retina to reattach	4 (33.3%)
**Complications**	
«Fish eggs» with subretinal gas	0
Vitreous hemorrhage	0
Elevated IOP	0
Traumatic cataract	0
Endophthalmitis	0
Cystoid macular edema	0
Macular hole	0

Legend: IOP = intraocular pressure

**Table 4:** Comparison of patients with and without primary anatomic success rate after PnR

Demographic data (*n* = 76)	Primary reattachment (*n* = 64)	Failure (*n* = 12)	*P* value
Age, mean ± SD	57 ± 6.6	72 ± 3.2	**< 0.001**
Female, number (%)	36 (56.2%)	4 (33%)	0.209
BCVA (LogMar), mean ± SD	0.2 ± 0.2	1.1 ± 0.3	**< 0.001**
FOVEA OFF, number (%)	12 (18%)	12 (100%)	**< 0.001**
Phakic patients, number (%)	56 (87%)	12 (100%)	0.342
Number of retinal breaks, mean ± SD	1.5 ± 0.6	1 ± 1.8	0.059
Extension of retinal detachment (hours), mean ± SD	3.0 ± 1.2	5.6 ± 1.8	**< 0.001**
Time to treatment (hours), mean ± SD	20 ± 11.3	32 ± 12.2	**0.032**
Subsequent PPV, number (%)		12 (100%)	

Legend: SD = Standard Deviation. BCVA = best corrected visual acuity. PPV = pars plana vitrectomy

**Table 5:** NEI VFQ-25 scores at 6 months for all patients treated with PnR

NEI VFQ-25 (*n* = 76)	Overall scores	
	Mean (SD)	Median (IQR)
Composite score	93.96 (6.4)	96.1 (92.8–97.7)
General health	69.23 (20.3)	75 (50–75)
General vision	77.30 (19.86)	80 (75–85)
Ocular pain	91.53 (10.41)	90 (87.5–100)
Near activities	97.56 (6.88)	100 (100–100)
Distance activities	94.21 (13.49)	100 (91.6–100)
Social Functioning	97.91 (7.05)	100 (100–100)
Mental Health	83.65 (18.79)	87.5 (81.2–93.7)
Role difficulties	96.15 (9.19)	100 (100–100)
Dependency	99.35 (2.28)	100 (100–100)
Driving	98.06 (4.9)	100 (100–100)
Color vision	100	100 (100–100)
Peripheral vision	98.07 (6.8)	100 (100–100)

Legend: NEI VFQ-25 = 25-Item National Eye Institute Visual Function Questionnaire. SD = standard deviation, IQR = interquartile range

## Discussion

Pneumatic retinopexy is a minimally invasive, and cost-effective procedure to repair primary RRD. The main finding of the present study was that patients selected and treated with PnR, according to the criteria and the surgical indications of the PIVOT trial, achieved a primary anatomic reattachment rate of 84% at 6 months. Moreover, in those patients in which PnR failed and underwent a subsequent PPV with cataract extraction and IOL implantation, the final anatomic reattachment rate was 100%. The results of the present study are in line with those reported in the PIVOT trial, where the primary anatomic reattachment rate was 81% in patients treated with PnR with a final anatomic reattachment of 98% [8]. To the best of our knowledge, this is the first study to report the outcomes of PnR in treating RRD in a real-world setting, using the surgical indication and the inclusion criteria of the PIVOT trial. Previous studies reported a single procedure anatomic reattachment rate in patients treated with PnR ranging from 40 to 93%, and a final anatomic reattachment rate ranging from 92 to 100% [18].

Another result of the present study was that the mean time between diagnosis to treatment was 22 h (minimum of 3 h - maximum of 48 h), whereas in the PIVOT trial, the mean time from randomization to PnR was 2 h (interquartile range 1–4 h). It is worth noting that in the PIVOT trial, PnR was performed in the office, whereas in the present study PnR was performed in the operating room. Our data suggests that PnR, even when performed in the operating room, with a longer interval between diagnosis and treatment, it is successful in achieving excellent anatomic and functional outcomes.

In the present study, the main cause of surgical failure was related to additional (most likely missed retinal breaks) (66.6% of failures), in line with the results reported in previous studies [19]. These additional retinal breaks were detected in eyes with persistent retinal detachment after PnR in the post-operative ophthalmological assessment. In cases of primary surgical failure, PPV in combination with phacoemulsification and IOL implant was performed, and none of the patient was treated with a second bubble injection. Overall, final anatomical reattachment was achieved in every patient (100%). Moreover, we observed that primary surgical failure was more frequent in eyes of patients with older age, extent of retinal detachment, foveal involvement, worse BCVA at presentation and increased time from diagnosis to treatment. There are two main mechanisms responsible for reattaching the retina after the injection of gas: the gas bubble tamponades the retinal break, allowing the RPE pump to reabsorb the SRF; the steamroller technique allows the gas bubble to roll along the detached retina, exerting a significant buoyant force to the retina, expressing the SRF through the retinal break back into the vitreous cavity. Previous studies reported that patients older than 60 years old, pseudophakic eyes, patients with a worse baseline BCVA, patients with a greater extent of retinal detachment and male gender were among the main risk factors associated with an increased risk of failure after PnR [19, 20]. In accordance with previous studies, we found that older age, extent of retinal detachment and worse baseline BCVA were more frequent in eyes of patients that presented surgical failure. Moreover, we found that macula involvement was more frequent in eyes of patients that presented surgical failure, as 100% of failures occurred in patients with a macula-off RRD.

In accordance with the criteria of the PIVOT trial, we included patients over 18 years old without setting an upper age limit. Previous studies have indicated that older age might increase the risk of failure in PnR [19]. In the elderly, the increased risk of surgical failure after PnR could depend on several factors, including difficulties in maintaining the required post-operative position, the pseudophakic status, and the presence of multiple small peripheral retinal breaks that are harder to detect pre-operatively [21]. A recent retrospective study by Muni and colleagues reported a primary anatomic reattachment rate of 78% after 3 months in patients over 75 years old treated with PnR, a rate comparable to outcomes achieved with other surgical techniques, such as pars plana vitrectomy and scleral buckle [22]. Thus, we believe that further studies are needed to compare the outcomes of PnR performed according to the PIVOT trial across different age groups, including a comprehensive assessment of the concomitant ocular and systemic comorbidities.

Furthermore, we observed that patients with surgical failure due to additional retinal breaks presented a more extensive retinal detachment and a lower visual acuity at the pre-operative assessment compared to those whose failure was due to poor compliance. (6.5 ± 1.6 vs. 4.0 ± 0.1 h, *p* = 0.012; 1.30 ± 0.12 vs. 0.70 ± 0.11 logMar, *p* < 0.001). A comprehensive pre-operative assessment that includes a scleral-depressed peripheral retinal examination is essential for identifying patients suitable for PnR. However, identifying small and peripheral breaks in patients with extensive retinal detachment is challenging due to the obscured view caused by retinal elevation. Hence, the results of our study suggest that patients with extensive retinal detachment are more likely to experience a surgical failure after PnR due to the presence of additional and most likely missed retinal breaks.

Another result of the present study was that BCVA significantly improved in patients with fovea-off RRD treated with PnR at 6 months, with a final BCVA of 0.04 LogMar, comparable with the final BCVA in patients with fovea-on at the time of surgery (0.03 LogMar). These results suggest that PnR is effective in restoring visual acuity in patients with a fovea-off RRD, even though this group of patients may present a higher risk of failure compared to patients with fovea-on RRD. Subjective visual function was assessed at the 6 month visits using the NEI VFQ-25, a validated quantitative questionnaire that has been used to assess patients’ vision-related quality of life in various ophthalmologic diseases and interventions [23, 24]. In the present study, the mean composite score was 93.9% at 6 months, similar to the mean composite score of 89% at 6 months reported in the PIVOT trial [9]. Previously, Muni et al. reported that patients undergoing PnR for RRD reported higher mental health scores and superior vision-related functioning scores in several subscales of the NEI VFQ-25 questionnaire during the first 6 months postoperatively compared with PPV, with no differences at 12 months [9]. Moreover, a recent study from their group reported a higher risk of discontinuity of the ellipsoid zone (EZ) and external limiting membrane (ELM) at 12 months postoperatively in patients treated with PPV compared to PnR for RRD repair, suggesting that less discontinuity of the EZ and ELM may provide an anatomic basis for the reported superior functional outcomes with PnR [25].

Complications with PnR are uncommon and generally resolve spontaneously [26]. The intra-operative complications that can occur during PnR include “fish eggs” formation with subretinal gas, injection of gas in the anterior hyaloid or in the suprachoroidal space, vitreous incarceration in the injection tract and vitreous hemorrhage [27]. Post-operatively, PnR can determine an increased IOP, formation of PVR, cystoid macular edema, macular folds, macular hole, cataract and endophthalmitis [27, 28]. In the present study we did not encounter minor or major complications in the patients treated with PnR. In the PIVOT trial, 7 patients developed cystoid macular edema (CME) and one patient developed bacterial endophthalmitis [8]. Both randomized trials related to PnR did not show a significant increase in risk of PVR, and this is likely related to the timely management of patient who are failing PnR [6, 8]. Also, IOP issues are also extremely rare with PnR, with most patients having normal IOP the following day [27].

The primary advantages of pneumatic retinopexy (PnR) are the reduced tissue trauma and the short time required to perform the surgery. Also, PnR avoids the need for hospitalization, making it a more cost-effective procedure compared to PPV and scleral buckle [29, 30]. Besides these benefits, PnR have shown to offer additional structural and functional advantages, contributing to better overall treatment outcomes [9, 25]. On the other hand, PnR is not suitable for all types of retinal detachments, particularly those with multiple or inferior breaks. Also, it requires strict post-operative head positioning, which can be challenging for patients to maintain. Additionally, PnR has a lower primary reattachment rate compared to PPV (71–96.7%) and scleral buckling (68.2–93.7%), potentially necessitating further procedures [31]. Overall, the results of our study suggest that using the criteria and the surgical indications of the PIVOT trial, PnR is likely to succeed in treating selected patients with primary RRD with excellent anatomic and functional outcomes, even in the real-world setting.

The main limitation of the present study is its retrospective nature. Moreover, we included a small cohort of patients, with a short follow-up of 6 months. However, in the PIVOT trial, it was demonstrated that patients who achieved retinal reattachment at 3 months are unlikely to subsequently re-detach. Future studies will be necessary to assess the primary anatomic reattachment rate, the final reattachment rate, the subjective visual functions, and the rate of late recurrences over a longer follow-up, using the criteria and surgical indication of the PIVOT trial in the real-world. Moreover, additional studies are required to elucidate the differences in NEI VFQ-25 scores between eyes with fovea-on and fovea-off detachments after PnR, as well as between eyes of patients who experienced primary or secondary retinal reattachment, and among different age groups.

To conclude, the results of the present study suggest that even in a real-world setting in Italy, where the use of PnR has been extremely limited, by using the surgical indications and the inclusion criteria of the PIVOT trial, PnR is likely to succeed in treating selected patients with primary RRD with excellent anatomic and functional outcomes. Therefore, PnR should be considered as a first line treatment in those patients that fulfill the inclusion criteria of the PIVOT trial.

## Data Availability

The data that support the findings of this study are available on request from the corresponding author.
